# Supplementary material for: Are carbon emissions trading and green financial instruments synergistic? -Comprehensive quantitative research based on content analysis
Source: PLoS One. 2024 Mar 7;19(3):e0298601. doi: 10.1371/journal.pone.0298601 (PMC10919601; doi:10.1371/journal.pone.0298601)
Supplement: S1 Data — (ZIP) [file pone.0298601.s001.zip › Data/地级市Z_F工作报告.zip!/░▓╗╒/╗┤▒▒╩╨/└┤╫╘╣1⁄2╓┌║┼í╛┬φ┐╦╩2╛▌═°í┐_2015.docx]

各位代表：

    现在，我代表市人民政府向大会报告工作，请予审议，并请市政协委员和其他列席人员提出意见。

    ****一、凝心聚力，攻坚克难，精致淮北建设迈出坚实步伐****

    2014年是我市改革发展进程中极不平凡的一年。面对严峻形势和繁重任务，全市人民在中共淮北市委的坚强领导下，认真贯彻党的十八大和十八届三中、四中全会精神，围绕建设精致淮北目标，应对经济下行压力，保持转型发展定力，迎难而上，逆势前行，经济社会持续平稳发展。预计全市实现生产总值750亿元，增长9%；财政收入91.35亿元，下降1.8%，其中地方财政收入52.81亿元，增长4.2%；固定资产投资833亿元，增长19%；社会消费品零售总额219.8亿元，增长12.9%；进出口总额5.4亿美元，增长16%；城镇居民人均可支配收入23400元，增长8%，农村居民人均可支配收入10500元，增长11.5%；居民消费价格涨幅1.3%；城镇新增就业4.2万人，城镇登记失业率4.06%；节能减排完成省控目标。

    一年来，我们以“九个一工程”为抓手，统筹推进各项重点工作，一批事关长远和全局的大事取得重大进展。交通建设完成投资18亿元，为历年之最。煤化工快速通道、濉唐路及合相路改建工程加快推进，一批损毁严重的省县道完成整修。浍河南坪港全面建成通航。经过不懈努力争取，全市人民期盼已久的高铁项目正式开工建设。安徽（淮北）新型煤化工合成材料基地建设上升为省级战略，焦化二期4个关联项目进展顺利。海聚科技园一期、市职教园区一期工程建成投用。4.92平方公里的南湖核心景区建成并向市民开放。淮水北调市级配水工程规划编制完成，华家湖水源涵养与生态补水工程初显成效。濉溪经济开发区积极争创国家级开发区，市、县经济开发区和凤凰山经济开发区获评全省投资环境十佳开发区。坚持不懈开展文明创建活动，获评首届安徽省文明示范城市，并再次被推荐为全国文明城市提名城市。

    一年来，我们主要做了以下工作：

    ****一是着力深化改革开放，发展活力不断增强。****坚持以改革创新破解难题，以扩大开放促进发展。改革行政审批制度。市级行政审批事项由390项减少到253项，精简35.1%。改革工商注册资本登记制度，有效激发了市场主体活力，全市新注册企业3301户，增长52.4%，民间投资占比上升到76.8%。推进财税金融改革。调整市、区分税制财政体制，充分调动区级科学理财、加快发展的积极性。改革预算编制管理，全面公开预决算和“三公”经费信息。扩大营业税改增值税试点。湖商村镇银行、濉溪农商行和淮矿集团财务公司挂牌运营。推动社会领域改革。顺利完成工商质监食品药品监督管理体制改革。探索教育管办评分离改革，推进职业教育市级统筹。巩固扩大基层医改和县级公立医院综合改革成果。稳步推进户籍制度改革，新发放居住证5846张。创新城市建设管理体制机制。通过政府购买服务，城区1000万平方米环卫保洁作业顺利实现市场化运作。成立市投融资委员会，完善政府性融资平台功能，引导更多社会资本参与城市基础设施建设。积极探索房屋征收货币化安置。深化农村综合改革。农村土地承包经营权确权登记颁证试点工作稳步推进，完成土地确权登记颁证面积67万亩。全面推进小型水利工程建管体制改革，改造提升“九小水利”工程，打通农田水利“最后一公里”。推进林权抵押贷款，建立公益林补偿机制。持续推进招商引资。市级领导牵头成立17个招商团，大力开展组团式协同招商、委托招商、以商招商，遴选39名年轻干部挂职招商。引进并开工投资规模亿元以上项目105个，迎辉光电、金富士食品等一批重大招商项目落户淮北。组织开展了重点项目集中开工活动，三本车业、恒兴汽配等44个项目开工建设，其中28个项目实现当年引进、当年签约、当年开工。七个省级开发园区完成基础设施投资8.2亿元，新增建成区面积6.6平方公里，引进亿元以上工业项目65个。

    ****二是着力应对困难挑战，经济保持稳定增长。****面对经济下行压力，采取扩投入、促消费、强实体等综合举措，确保经济运行平稳有序。加大项目投资。强化市级领导联系督查重点项目制度，加快推进427个重点项目，完成投资490.9亿元，其中153个工业项目完成投资191.8亿元，增长25.1%。相邦铝基复合材料、银龙三佳高铁轨道板、华中天力铝业、思朗保健食品、中威矿山设备等一批重大产业项目相继建成或竣工投产。平山电厂累计完成投资15.4亿元。持续扩大消费。完善城市商业网点布局，全市5000平方米以上大型商业网点增至44个。完成相山路标准化菜市场维修改造，建成乡镇商贸中心9家，直营农家店120家。推动电子商务发展，淮北企业网开通运营。加快发展流通产业，新增限额以上商贸流通单位107家。出台促进房地产市场平稳健康发展的意见，完成房地产投资152亿元，销售商住房面积172万平方米。全力帮扶企业。免征开发园区企业38项行政事业性收费，减轻企业负担3300万元。缓缴和减收企业社保费11.24亿元，发放稳定岗位和技能培训补贴4814万元。强化市企战略合作，支持省属煤炭企业深化改革、转型发展。深入开展“干部帮联企业，打造精良产业”行动，扶持小微企业发展，新增规模以上工业企业89家，总数达739家，规模以上工业增加值540亿元，增长11%。强化要素保障。加强政银企对接合作，各项贷款余额664.2亿元，较年初增加50.5亿元。扩大企业债券发行规模，直接融资94亿元，10家企业签订“新三板”、“四板”合作协议。多途径保障发展用地，上报获批各类建设用地13692亩，清理闲置土地1950亩。

    ****三是着力推进转型升级，结构调整取得积极成效。****三次产业比例由上年8.1：67.2：24.7调整为8：66.9：25.1。现代农业稳步发展。粮食生产实现“十一连丰”，成为全省第二个小麦亩产千斤市。农业产业化水平不断提升，规模以上农产品加工业产值510亿元，增长16.5%。“段园葡萄”成为国家地理标志产品。新增土地流转面积3万亩，家庭农场增至861家，农民合作社发展到1164家。工业结构持续优化。机械制造、食品工业、纺织服装和建材业等非煤产业完成增加值345.5亿元，占全市工业的64%，同比提高6.4个百分点。电子信息、铝基新材料等战略性新兴产业提速发展，实现产值407.7亿元，增长20.2%。煤化工产业链进一步延伸，首次实现全线盈利。服务业发展步伐加快。出台加快发展物流业的指导意见。淮北汽车城获批省级服务业集聚区。国购城市综合体、中泰广场基本建成，淮矿物流园二期、中易物流园和国购汽车文化园、隋唐运河古镇、政通梦幻王国等项目加快建设。成功举办第三届食品工业博览会和第五届石榴文化旅游节。服务业占全市生产总值比重同比提高0.4个百分点。科技支撑作用不断增强。新增授权发明专利113件，增长22.8%，新认定国家高新技术企业25家。高新技术产业产值突破400亿元，增速居全省前列。相邦复合材料创新团队列入省高层次科技人才团队计划。龙湖高新区获批设立省级“留学人员创业园”。杜集矿山机电装备科技企业孵化器获省级认定。烈山国家可持续发展实验区、凤凰山国家级农业科技园通过科技部现场评审。

    ****四是着力建设精美城市，城乡统筹展现新面貌。****坚持建管并举，突出环境整治，进一步提升城市形象。持续完善城市功能。投资16.3亿元，实施城市道路、管网、桥梁、绿化和电网建设等重点项目127个。维修加固东岗楼立交桥，新建、改造东山北路、惠黎路、长山中路等14条城区道路，东部新区4条道路建成通车。增设公交候车亭54座，更新改造人民路、相山中路路灯697柱，完成长山路桥等7座道路桥梁和部分街区广场亮化工程。推进供水、供气向城市周边延伸，新铺设燃气、供水和污水管网84公里。完成2570公里地下管线普查探测并建成信息管理平台。实施城市增绿工程，建成绿道34.3公里。顺利通过国家园林城市验收。整治老濉河、相阳沟、跃进河等城区沟河，清理淤泥24万立方米。完成房屋征收191万平方米，建成安置房89万平方米，已有3000余户居民迁进新居。强化城市管理服务。加强“市容管理示范路”建设，完成濉溪路等6条道路街景整治。实施公共自行车惠民工程，日均使用量4.5万人次。出台进一步加强物业管理工作的实施意见，规范物业管理。生活垃圾无害化处理率达94%以上，入选国家第四批餐厨废弃物资源化利用和无害化处理试点城市。国家智慧城市试点建设取得积极进展。加快建设美好乡村。首批13个重点中心村全面建成，统筹推进第二批11个重点中心村规划建设。深入开展“三线三边”治理，农村人居环境不断改善。濉溪县获评全省美好乡村建设先进县，烈山区入选全国美丽乡村建设标准化试点，榴园成为全省宜居村庄示范村。注重生态环境保护与建设。加强工业大气污染防治和机动车排气污染治理，严控污染物排放增量，改造燃煤锅炉68台，淘汰黄标车、老旧车8659台。定期开展采石场及石料加工、物料堆场、渣土运输、建筑工地、混凝土搅拌站等环境专项整治。积极抓好秸秆禁烧和综合利用工作。组织实施节能降耗专项行动，推进绿色循环低碳发展。全力争创全国绿化模范城市，完成森林增长工程造林 6.6万亩、森林长廊建设66.7公里、东部山场绿化1.6万亩，全市森林覆盖率达20.5%。全面推进东湖及华家湖周边采煤塌陷地、废弃矿山等地质环境整治与生态修复，完成治理面积3196亩。

    ****五是着力保障和改善民生，社会建设进一步加强。****在收支矛盾较为突出的情况下，用于民生领域支出92.5亿元，占财政总支出的80.7%。民生工程全面完成。34项民生工程投入29.1亿元，比上年增加7.6亿元，群众得到更多实惠。就业和社会保障工作扎实推进。坚持以创业带动就业，突出抓好重点群体就业。积极推进城镇居民大病医疗保险，进一步完善社会救助体系。城乡低保标准、企业离退休人员和被征地农民养老金稳步提高。两所敬老院、900张民办养老机构床位、64所社区养老服务站建成使用。发放家庭经济困难学生资助金5815万元，受益学生7万多人次。420户“以船为家”渔民全部上岸安居，“老字号”群体工龄补助发放政策严格落实。实施公租房廉租房并轨运行，新开工保障性住房和棚户区改造住房1.35万套，基本建成1.2万套，710户农村危房改造工程全部竣工。发放公积金贷款23亿元，10470户家庭住房条件得以改善。社会事业加快发展。推进城区教育资源合理配置，整体提高薄弱学校办学条件，市辖三区全部通过国家义务教育均衡发展验收认定。新农合工作在全省率先推行二代身份证读卡结算报销，并实行按床日付费。柳孜隋唐运河遗址跻身世界文化遗产名录，成为我市一张世界级文化名片。文化惠民基础设施逐步完善，公共文化服务场所全部免费开放。我市在省十三运会、六届残运会和七届民族运动会上取得较好成绩，八一男排全国联赛主场落户淮北。社会大局和谐稳定。完成15个社区综合服务中心、12个城市标准化示范社区、30个村级组织服务用房建设。276个村顺利完成新一届村民委员会换届选举工作。我市跻身全国首批社会组织建设创新示范区。扎实开展平安淮北建设，坚持社会治安综合治理，群众安全感指数同比上升3.1个百分点。市政府应急指挥信息系统投入使用。稳步推进信访工作制度改革，基层综治信访维稳工作平台全面建成。开展清理拖欠农民工工资专项行动，切实维护农民工合法权益。安全生产实行“党政同责、一岗双责”，积极开展打非治违和隐患治理，关闭地方小煤矿8对，淘汰落后产能92万吨，各类事故和死亡人数实现双下降。严厉查处制售假冒伪劣食品药品行为，保障人民群众饮食用药安全。圆满完成战时国防动员演练。第三次经济普查工作荣获全国先进。妇女儿童、青少年、老龄、残疾人、红十字等事业取得进步，人口和计划生育、司法行政、双拥共建、人防、民族宗教、外事侨务、对台、气象、地震、档案、保密、地方志等工作继续加强。

    各位代表！在推进经济社会发展的同时，我们注重加强政府自身建设，不断提高运用法治思维和法治方式推动科学发展的能力。深入开展党的群众路线教育实践活动，认真查摆“四风”方面的突出问题，对自查和征求到的475条意见建议列出“问题清单”，逐项逐条分解，狠抓整改落实和建章立制。坚持依法行政，规范政府系统重大决策行为。自觉接受人大法律监督、政协民主监督和社会舆论监督，办理市人大代表议案、建议133件和市政协委员提案186件，满意度99%以上。办好《政风行风热线》、《说办就办》和《法制时空》栏目，办理市长信箱和网上留言1358条，累计为群众解决实际问题4512个。严格落实中央八项规定、国务院“约法三章”，修订完善市政府工作规则，强化行政监察、审计监督和政务督查，务实改进政府作风。精简会议文件，清理超标准办公用房，规范公务接待，严控“三公”经费支出，勤政廉政建设进一步加强。

    各位代表！过去的一年，我们遇到的困难超乎预期，付出的努力非同寻常。全市上下在挑战中坚定信心不动摇、改革攻坚求突破，经受住了严峻考验，取得了来之不易的成绩。这是市委正确领导、科学决策的结果，是全市人民众志成城、共克时艰的结果。在此，我代表市政府向全市人民，向人大代表、政协委员，向各民主党派、工商联、无党派人士和人民团体，向各位老领导、老同志，向驻淮解放军、武警官兵和政法干警，向国家和省驻淮单位，向在我市创业的投资者、建设者，表示诚挚的感谢!

    在看到成绩的同时，我们也清醒认识到经济社会发展中还存在不少困难和问题。主要是：经济下行压力依然较大，部分骨干企业和小微企业生产经营困难、效益下滑。新兴产业规模不大，服务业发展滞后，招商引资氛围不浓、大项目偏少，财政收支矛盾突出，社会治理任务繁重。少数公职人员工作作风不实，服务和担当意识不强；公职人员当中自我要求不严、不勤不廉现象时有发生。对此，我们将坚持问题导向，采取积极措施，有效加以解决。

****二、适应新常态，把握新机遇，奋力推动经济社会持续稳步发展****

    2015年是全面深化改革的关键之年，是全面推进依法治市的开局之年，也是全面完成“十二五”规划的收官之年，做好今年政府工作，任务艰巨、意义重大。从宏观形势看，世界经济仍处在国际金融危机后的深度调整期，总体复苏疲弱态势难有明显改观；我国经济进入中高速、优结构、新动力、多挑战为主要特征的新常态，发展条件和环境发生深刻而复杂的变化。从淮北自身看，受煤炭行业产能过剩、需求萎缩、价格下跌等当前因素，以及我市产业结构不优、经济总量不大、质量效益不高等长期累积问题影响，经济运行仍将处于非常困难时期。必须从当前经济发展的阶段性特征出发，积极适应新常态，既要看到宏观经济减速换挡、转方式调结构更加紧迫，更要看到我市改革活力增强、经济增长动力正从以煤为主转向多点支撑、民营经济加快发展、一大批项目正在加紧建设等利好因素，继续保持战略定力和历史耐心，以改革创新的思路、开放发展的举措、转型升级的成效，赢得未来发展的主动。

    ****今年政府工作的总体要求是：****全面贯彻党的十八大、十八届三中、四中全会和中央、全省经济工作会议精神，坚持稳中求进工作总基调，紧紧围绕建设精致淮北目标，主动适应经济发展新常态，以提高经济发展质量和效益为中心，以深化改革开放为动力，以调结构促转型为主攻方向，以推进自主创新和发展民营经济为重要驱动，着力保障和改善民生，强化风险防控，全面推进依法治市，促进经济持续健康发展和社会和谐稳定。

    ****经济社会发展主要预期目标是：****全市生产总值增长8%左右，财政收入增长1.1%，固定资产投资增长13%，社会消费品零售总额增长11%，外贸进出口总额增长11%，城镇居民人均可支配收入增长8%，农村居民人均可支配收入增长11%，城镇新增就业4.2万人，城镇登记失业率控制在4.2%以内，节能减排完成省定任务。

    重点做好十个方面工作：

    ****（一）着力推动重要领域改革****

    坚持以改革促进稳增长、调结构、惠民生，积极有序推进重点领域和关键环节改革。深化行政体制改革。实施政府机构改革，科学配置部门职能，理顺部门职责关系。承接落实好国务院和省取消、下放的行政审批事项，继续精简市级行政审批项目。公布政府及其工作部门权力清单、责任清单和涉企收费清单，完善事中事后监管机制。推进政务服务标准化建设，实行行政审批“两集中、两到位”。实施办公用房管理和公务用车制度改革。加快事业单位分类改革。深化财税金融改革。创新政府融资平台运行机制，深化土地收储和投融资管理体制改革。改革财政支持企业发展方式，建立产业投资基金，重点支持战略性新兴产业和现代服务业发展。将政府性债务收支纳入预算统一管理，加强风险防控。充分发挥综合治税平台作用，坚持依法征管。全面落实资源税、营改增等税改政策。大力引进域外银行来淮设立分支机构。扩大企业直接融资渠道和规模，重点推进中小企业在场外市场挂牌融资。深化城市规划建设管理体制机制改革。按照统分结合、责权利一致原则，明确界定市、区在规划建设管理方面的事权和责任，建立科学合理、运转高效的城市管理体制。实施招投标管办分离体制改革，建立“一委一办一平台”运行机制。深化城乡发展一体化综合改革。加快推进农村土地确权登记颁证工作，落实农民对承包地占有、使用、收益、流转及承包经营权抵押、担保权能。创新水利发展体制机制，继续做好相山区机井管护和刘桥水厂建管试点工作。推广政府与社会资本合作（PPP模式），鼓励社会投资参与基础设施建设运营。探索市场化的淮水北调市级配水工程投融资体制、建管体制和水价形成机制。建立公共资源城乡均衡配置体制，促进城乡基本公共服务均等化。积极争取国家中小城市综合改革试点。深化文化和社会事业改革。鼓励社会力量、社会资本参与公共文化服务体系建设。推行县区义务教育学校教师无校籍管理，推进职业教育向多元投入、产教融合、校企合作方向发展。积极推进市级公立医院改革，巩固完善基层医改和县级公立医院综合改革。深化收入分配制度改革。稳步实施机关事业单位养老保险制度改革。

    ****（二）全力抓好招商引资和项目建设****

    推动淮北转型发展、加快发展，关键靠投资拉动，靠招商引资，靠项目支撑。抓招商引资添增量。瞄准重点地区、重点领域、重点企业开展精准招商，抓好产业延链补链招商，引导外来投资更多地投向新型煤化工、食品加工、装备制造和铝基新材料、电子信息、节能环保等重点产业及现代服务业，提升招商引资产业符合度和投资强度。实施专题推介招商、委托招商和驻点招商，鼓励各载体单位依托标准化厂房、低效闲置土地等开展“零土地招商”和“二次招商”。加强与央企、知名民企对接，加大对苏浙沪、闽粤和港台招商力度，推动区域经济合作，全年引进并开工规模以上项目100个。抓项目建设增后劲。今年计划安排重点项目435个，完成年度投资453亿元。强化市级领导联系督查重点项目制度，完善项目协调推进机制，突出抓好“九个一工程”建设。继续开展“抓在建、促投产”行动，全面提升项目开工率、竣工率和投产率。推动平山电厂2台66万千瓦机组年内投产运行，积极推进国安电力二期、中利电厂三期及大唐热电联产项目前期工作。合理确定213个政府性投资项目，带动和激活更多的市场主体增加投入。抓园区提升促集聚。加快推进七个省级开发园区建设，做好市开发区新区产城一体化总体规划和龙湖高新区规划调整评审及报批工作，加快标准厂房、公租房和配套服务设施建设。完善临涣工业园基础设施配套，积极推动与上海华谊、神华集团等大企业合作，促进煤化工产业上规模、提质效。继续推动濉溪经济开发区向国家级开发区迈进。支持杜集经济开发区打造国家级矿山机电装备高新技术产业基地和新型工业化产业示范基地，支持凤凰山开发区打造国家级食品产业基地，促进产业集聚发展。推动市开发区新区与烈山区5平方公里园中园竞相发展。加快濉芜现代产业园基础设施建设，尽快引进一批工业企业入园发展。

    ****（三）创新驱动工业结构优化升级****

    坚持调整存量与做优增量并举，进一步强化创新驱动发展的引擎作用，依靠创新培育工业竞争新优势。改造提升传统产业。加强信息化和工业化融合，加快技术创新与技术改造，促进传统产业创新产品、创新管理、创新商业模式。进一步巩固和发展共生共赢的市矿关系，共同谋划和实施煤电、煤化联产项目。抢抓电力体制改革的机遇，争取获批直供电和自备电。积极推进新型煤化工合成材料基地建设，确保年内焦化二期、年产20万吨混合芳烃加氢项目建成投产，推动宝钢气体空分装置、干熄焦余热发电、煤焦油深加工、特种炭黑等项目开工建设，不断延伸煤电化产业链。依托安徽矿机、口子酒业、华孚色纺等骨干企业，抓好关键技术和装备的改造提升，重点推进138个工业转型升级技改项目。深入开展“干部帮联企业、打造精良产业”行动，做大做强行业领军企业，加快专精特新企业成长，促进小微企业“小升规”，全年新增规模以上工业企业80户，规模以上工业增加值增长9%。着力培育壮大新兴产业。坚持把培育发展战略性新兴产业作为调结构、促转型的重大举措，强化政策支持，推进电子信息、铝基新材料、生物医药、节能环保和高端装备制造等5大战略性新兴产业规模化发展。加快建设傲龙网络、大华活性炭等24个投资亿元以上新兴产业重点项目，确保战略性新兴产业产值增长15%。大力提升自主创新能力。实施科技提升园区、产学研协同创新、科技型企业培育行动计划，深化与中科院、上海交大、淮北师大等高校与科研院所合作，抓好工程技术研究中心、企业技术中心建设。新增国家高新技术企业15家，实现高新技术产业产值460亿元。启动国家知识产权示范市培育工作。争创全国质量强市示范城市。围绕经济结构调整和新兴产业发展，加大创新人才培养引进力度。

    ****（四）促进服务业发展提速增效****

    坚持把发展服务业与提升城市功能、优化产业结构结合起来，完善鼓励服务业发展的政策措施，支持在淮省属企业发挥自身优势，在稳定主业的同时，拓展现代服务业；引导各类社会资本平等进入服务业领域，开发多层次市场，促进服务业消费。优先发展生产性服务业。力争在研发设计、电子商务、物流快递、节能环保服务及品牌建设等方面有大的突破。加快电子商务平台建设，拓展本地特色产品销售渠道。做好现代物流业发展规划修编工作，推进青龙山商贸物流园等规划建设。加强与上海铁路局和合肥海关合作，积极推动青龙山火车站“无水港”建设。促进金融等新型业态加快发展。升级发展生活性服务业。推进城市商业综合体、专业市场和商贸购物中心建设，提升核心商圈辐射功能。引导大型连锁超市、批发市场等流通业态转型升级。继续推进“万村千乡市场工程”、“农超对接”和“企超对接”工程。促进文化旅游体育融合发展，拉动体育健身消费，加快隋唐运河古镇、政通梦幻王国等文化旅游项目建设，谋划打造隋唐运河古文化、美好城乡自然风光和红色文化传统教育三大主题精品旅游线路，推动“四季榴园”、南湖创建国家4A级景区。创办“房产超市”，实现房地产市场消费常态化。加快发展新兴服务业。将智慧城市建设与发展新兴产业有机结合，促进各种创新要素聚集、交融，催生新的业态和商业模式，推动发展物联网、移动互联网、创意应用等智慧型新兴产业。大力发展信息消费、养老和健康服务业。发展假日经济、会展经济，办好2015中国淮北食品工业博览会、第六届石榴文化旅游节。

    ****（五）夯实农业农村发展基础****

    在稳定粮食生产基础上，大力推进农业结构调整，加快转变发展方式，不断提高农业综合效益。持续增加粮食等农产品供给。积极推进土地治理，完成高标准农田水利建设2万亩。继续开展粮食高产创建活动，建立135万亩小麦、40万亩玉米高产攻关核心示范区。实施“粮安工程”危仓老库维修改造。抓好“主食厨房”、“放心粮油”工程，助推食品工业快速发展。加强“菜篮子工程”建设，扩大蔬菜生产基地，发展规模养殖，培育100个标准化规模种养示范基地。加快创新农业经营体系。加大政策扶持力度，培育示范家庭农场50个，新增农民合作社示范社50家。健全农业社会化服务体系，做大做强龙头企业，带动各类经营主体共同发展。强化农业科技创新，推动农业物联网技术应用，加快五铺等6个现代农业示范园建设。完善口子等4个食品园区功能，推动食品加工向生物科技和保健养生方向拓展。努力争创凤凰山国家级农业科技示范园区。提升美好乡村建设水平。坚持从实际出发，因地制宜，量力而行，严防举债搞大拆大建，引导群众自觉自愿、投资投劳建设美好家园。结合“三线三边”环境治理，启动第三批11个重点中心村建设。以解决垃圾收集处理、安全饮水、道路畅通和改善生态为重点，带动全市村庄面貌逐步改善。大力实施兴业富民工程，推进“一村一品”、乡村旅游等特色产业发展，努力走出一条村庄建设与产业发展、群众致富互促共进的新路子。

    ****（六）大力发展民营经济****

    全面落实鼓励和支持非公经济发展的政策措施，不断深化改革、强化服务、优化环境，推动民营经济加快发展。激发市场主体活力。深化“三证合一”登记制度改革，推行“负面清单”管理模式和项目审批收费“一表制”，清理不必要的证照和资质、资格审批，降低企业创办门槛，全年新增私营企业2150户、个体工商户4800户。鼓励大众创业。依托现有创业孵化基地，支持大学生和海外留学人员领办、创办企业，引导更多外出务工人员回乡创业，促进更多个体工商户升级为企业法人，充分激活全社会的创业激情，努力在全市形成大众创业、万众创新的良好氛围。助推民营经济转型发展。发挥产业投资基金引导作用，鼓励和支持有实力的民营企业立足自身优势，把握市场需求，积极从传统产业向战略性新兴产业提升，向现代服务业延伸，向现代农业拓展。支持民营企业与科研院所合作，加大科技投入，加快新产品开发。提高企业家素质，发挥企业家作用，提升企业经营管理水平。着力优化发展环境。坚持以服务促管理、以扶持促发展，简化办事手续，提高服务效能。坚持依法行政，严肃查处滥用行政权力干预民营企业合法生产经营活动，让企业家放心、放胆发展。加强全社会诚信建设，以良好的诚信环境促进民营经济健康发展。建立银政担合作和代偿按比例分担机制，放大担保功能，为民营企业创造宽松的融资环境。

    ****（七）积极推进新型城镇化****

    坚持走以人为本、四化同步、优化布局、生态文明、传承文化的新型城镇化道路，稳步提升城镇化质量和水平。加强城市规划建设管理。实施全域城镇化战略，加强规划协同，分轻重缓急编制市区各区块修建性详规，做好城镇规划与土地规划衔接，编制土地储备规划，提高城镇土地利用率。启动高铁新区建设，完善东部新城区基础设施和商业配套，增强新城区对人口的吸引力。打通古城东路、环山东路、方安南路等城市断头路，加快龙山南路、桂苑东路建设，大力推进老城区主次干道、背街小巷、地下管网等基础设施改造，实施孟山南路贯通工程。建设烈山片区污水传输一期工程。建成南黎公园。以文明创建为抓手，持续加强城市环境综合整治，不断提升城市形象和管理水平。强化城市管理“一盘棋”意识，实行市区联动、部门协作、重心下移，充分发挥社区管理作用。深化“市容管理示范路”创建工程，完善网格化管理平台。新投放公共自行车3100辆。深化环卫作业市场化改革，进一步提升垃圾无害化处理和资源化利用水平。推行房屋征收货币化安置。全面提升城市物业管理水平。完善区域综合交通体系。加快淮北至萧县北高铁联络线工程进度，积极推进符夹铁路扩能工程和青阜铁路电气化改造工程。加快济祁高速淮北段、煤化工快速通道建设，完成合相路一期主体工程，续建萧淮路中段、濉唐路工程。开工建设南坪船闸，整治沱浍河航道。加强农村公路建设和养护管理，大力发展城市公交并向小城镇延伸，努力构建城乡互通、高速绕城、公铁水联运的综合交通网络，让淮北交通变得更加通达顺畅。加快县城和小城镇建设。按照满足城镇化快速发展需要并适度超前的原则，加大县城和小城镇市政公用设施建设力度，提升综合承载能力。推动天然气、集中供水等公共服务设施向城镇、乡村延伸。有序推进农业转移人口市民化。深化户籍制度改革，继续实行居住证制度，县城和建制镇落户全面放开。有效解决农业转移人口在城镇就业、就医、子女入学和社会保障等方面问题，让进城务工农民进得来、留得住、过得好。

    ****（八）持续加强生态文明建设****

    坚持污染防治和生态修复并重，构建资源节约型、环境友好型生态体系，打造“山青、水秀、天蓝、景美”的山水生态城市。强化污染防治。以创建国家环保模范城市为抓手，继续实施清洁生产、饮用水源地保护、总量减排、大气污染治理、固废处置和新能源替代等重点环保工程。加大扬尘污染防治和机动车尾气污染治理力度，专项整治煤炭、粉煤灰堆场和采石场，加强市区货运车辆通行线路管控。严格监测和控制城市PM10和PM2.5。继续推进火电、水泥等高污染行业脱硫、脱硝和除尘改造。加快污水集中处理工程及配套管网建设。下大力气抓好秸秆禁烧和综合利用工作。推进生态保护与建设。积极争创全国绿化模范城市，大力实施森林增长工程，绿化荒山4500亩，植树造林2.9万亩，争取今年让全市石质山披上绿装。推进城市主要道路、出入口和南湖公园、相山公园等重要节点景观绿化提升改造，新增城镇绿化170万平方米，建成绿道50公里。按照“调、输、蓄、用、节、净、美”的总体思路，统筹安排城市生产、生活、生态用水。加快推进淮水北调和市级配水工程，继续实施华家湖水源涵养和生态补水工程及城区水环境综合整治。抓好东湖、中湖采煤沉陷区治理和生态环境修复，治理废弃矿山2000亩。促进资源节约集约利用。做好工业、交通、建筑等重点领域节能工作，降低能源、水资源消耗。继续清理开发园区闲置及低效用地。促进循环经济发展，提高资源利用效率。

    ****（九）更加注重保障和改善民生****

    坚持以民生工程为抓手，推动社会事业全面协调发展。持续办好民生工程。全年计划安排财政资金30亿元，实施34项民生工程。继续提高义务教育公用经费、新农合和城镇居民医疗保险补助标准，将涉及困难群体生活的农村低保、五保供养等项目提标10%，扩大新农合大病保险实施范围以及贫困残疾人救助与康复救治面。完善绩效考评体系和建后管养机制，切实发挥民生工程的最大效益。积极做好就业和社会保障工作。抓好高校毕业生等重点群体就业，落实鼓励自主创业的扶持政策，促进创业带动就业。完善社会保险制度，推进医疗保险城乡统筹，全面实施城镇居民大病医疗保险，巩固扩大城乡居民社会养老保险制度覆盖成效。抓好社会保险扩面征缴，推进农民工参加社会保险。开工建设保障性住房和棚户区改造住房9600套，基本建成1.2万套（含续建项目），完成365户农村危房改造任务。统筹发展社会事业。促进义务教育均衡发展，继续实施学前教育三年提升计划。做好新建住宅小区同步配建幼儿园和义务教育学校工作。推进市职教园区、一中新校区、人民医院东院区建设。深入实施基本公共卫生服务项目，强化重大疾病预防控制。促进人口计生工作转型发展。推进柳孜隋唐运河遗址考古公园建设。加强基层公共文化体育设施管护，鼓励有条件的镇村建设农民文化乐园，完善全民健身公共服务体系。

    ****（十）努力保持社会和谐稳定****

    更加注重履行社会管理和公共服务职能，认真解决群众反映强烈的突出问题，确保社会安定有序。不断创新社会治理。扎实推进法治淮北建设。深入开展法治宣传教育，推进多层次多领域依法治理。完善公共法律服务体系建设，加强困难群体法律援助。健全社会矛盾纠纷预防化解机制，引导和支持群众理性表达诉求、依法维护权益，畅通和规范群众利益协调、权益保障渠道。完善初信初访办理制度，推进涉法涉诉信访改革。严格落实领导干部接访约访下访制度，推行“阳光信访”，全面实行网上受理信访制度。认真做好重大事项社会风险评估工作，从源头上预防和减少社会矛盾发生。全面夯实基层基础。以争创全国社会治安综合治理先进市为目标，大力加强社会治理基层基础工作。充分发挥基层综治维稳信访平台作用，进一步健全制度机制，落实保障措施。推进智慧城市和社会服务管理信息化建设，实现信息资源共享。深化城市社区网格化管理，加强社区矫正工作。完成开放式村级组织活动场所建设。加强公共安全防控。完善社会治安防控体系，严厉打击各类违法犯罪活动。健全应急处置联动机制，提高保障公共安全和处置突发事件能力。强化食品药品安全监管，深入开展“本质安全年”和打非治违专项活动，继续推进小煤矿关闭整顿工作，加大对重点行业和重点企业监管力度，依法严厉打击货运车辆超载超限运输，有效防范各类重大安全事故发生。加强国防、双拥和人防工作，支持驻淮部队建设，推进军民融合深度发展。

    ****三、强化法治理念，推进依法行政，建设法治政府****

    坚持把依法行政的要求贯穿于政府工作的始终，依法全面履行政府职能，加快法治政府建设进程。牢固树立法治思维。强化法治理念和依法行政意识，自觉运用法治思维和法治方式深化改革、推动发展、化解矛盾、维护稳定。健全依法决策机制。严格执行重大行政决策公众参与、专家论证、风险评估、合法性审查、集体讨论决定法定程序。坚持和完善听证制度、政府法律顾问制度。深化行政执法体制改革。完善市、县（区）行政执法管理，推进食品药品安全、工商质监、公共卫生、安全生产、文化旅游、资源环境、农林水利、交通运输、城乡建设等领域综合执法。坚持严格规范公正文明执法。强化权力监督制约。自觉接受人大法律监督、工作监督和政协民主监督，依法执行人大决议决定，重大决策出台前向人大报告，认真做好人大代表议案、建议和政协委员提案办理工作。广泛听取各民主党派、工商联和无党派人士意见，主动接受社会公众和新闻舆论监督，充分发挥工会、共青团、妇联等人民团体重要作用。加强对政府内部权力的制约，强化行政复议、行政监察和审计监督，更加注重审计成果的有效运用。全面推进政务公开。大力推进行政权力运行、财政预算、公共资源配置、社会公益事业建设等领域政府信息公开。推行行政执法公示制度。加强政府网站建设，推进政务公开信息化。

    各位代表！建设人民满意的政府，是人民群众的期盼，也是我们追求的目标。面对改革发展的新形势、新任务，全市各级政府工作人员必须积极适应经济发展新常态，始终保持奋发有为好状态。大力发扬“钉钉子”精神，真抓实干，勇于担当，以锲而不舍的韧劲推动各项工作落实。强化绩效考核和政务督查，严肃查处履职不到位、行政不作为、慢作为和乱作为等问题，着力提升行政效能。巩固和拓展党的群众路线教育实践活动成果，严格执行中央八项规定和国务院“约法三章”，严控“三公”经费支出，持之以恒反对“四风”，认真履行党风廉政建设责任制，自觉遵守廉洁从政各项规定，进一步树立为民务实清廉政府形象。

    让我们在中共淮北市委的坚强领导下，团结和依靠全市人民，以更大的决心、更足的干劲、更实的作风，开拓进取，克难奋进，为全面完成今年各项目标任务，为加快城市转型、建设精致淮北而努力奋斗！
